# The relationship between oxygen therapy, drug therapy, and COVID-19 mortality

**DOI:** 10.1515/med-2022-0569

**Published:** 2022-11-22

**Authors:** Ling Yang, Guoxi Chen, Yuyang Cai, Ye An, Xiaopan Li, Ying Chen, Cheng Xu, Chen Ji, Xing Lan, Yaling Wang, Hai Huang, Li Han

**Affiliations:** Department of Geriatrics, Shanghai Fourth People’s Hospital Affiliated to Tongji University, Shanghai, China; Tuberculosis department, Wuhan Pulmonary Hospital, Wuhan, China; Pudong Institute of Preventive Medicine, Fudan University, Shanghai, China; Warwick Clinical Trials Unit, Warwick Medical School, Warwick, Great Britain; School of Public Health, Shanghai Jiaotong University School of Medicine, Shanghai, China; Department of Geriatrics, Shanghai Jiaotong University School of Medicine, Xinhua Hospital, Shanghai, China

**Keywords:** drugs treatment, COVID-19, pneumonia, mortality

## Abstract

Since December, 2019, Wuhan, China, has experienced an outbreak of coronavirus disease 2019 (COVID-19). We conducted a retrospective study of COVID-19 inpatients in Wuhan Pulmonary Hospital (Wuhan, China) from January 1 to February 29, 2020. The subjects were divided into four groups due to different treatment regimes. We used the Kaplan–Meier method to determine the cumulative rates of in-hospital death and the Cox proportional hazard model to calculate the risk factors and corresponding hazard ratios. A total of 185 patients were included in this study. The median age of the patients was 62 years, including 94 men and 91 women. Kaplan–Meier analysis demonstrated that mortality was higher in older patients, higher in men, and lower in the low-flow oxygen therapy group. Body mass index (BMI) had no influence on mortality, as well as high flow oxygen therapy, Lopinavir–ritonavir (LPV/r) therapy, and the interferon-alpha add LPV/r therapy. Cox proportional hazard regression confirmed that the low flow oxygen therapy was independent protective factor for in-hospital death after adjusting for age, gender, and BMI. In conclusion, the mortality was higher in older patients, higher in men, and lower in the low-flow oxygen therapy group. BMI had no influence on mortality, as well as high flow oxygen therapy, LPV/r therapy, and interferon-alpha add LPV/r therapy.

## 1 Introduction

On December 31, 2019, the World Health Organization (WHO) was notified a cluster of cases of pneumonia with unknown etiology in Wuhan, Hubei Province, China. Chinese specialists rapidly isolated the novel coronavirus on January 7 and shared viral genome data with the international community [1]. The highly pathogenic virus is now called SARS-CoV-2 (initially called 2019-nCoV) [2]. The disease was named COVID-19 by the WHO [3]. With reports of thousands of new cases of SARS-CoV-2 infection in China and evidence of person-to-person transmission in the United States and other countries [4,5]; on January 30, the WHO declared the epidemic outbreak of a public health emergency of international concern. Specific treatment and prevention options, such as targeted antiviral drugs and vaccines, were not yet available. Several preexisting and potential drug candidates, including Lopinavir–ritonavir (LPV/r) and interferon-alpha, have been applied to the clinical antiviral treatment of the COVID-19. In this single-center retrospective study of COVID-19 adult patients in Wuhan Pulmonary Hospital, which was designated for COVID-19 cases by local government, we aimed to illuminate the relationship between different treatment regimens and inpatient all-cause mortality of COVID-19 patients.

## 2 Methods

### 2.1 Study design and participants

We included 185 patients with laboratory-confirmed COVID-19 from Wuhan Pulmonary Hospital (Wuhan, China), who had been discharged or had died by February 29, 2020. Laboratory diagnosis of SARS-CoV-2 is based on the positive viral nucleic acid test result on throat swab samples. Diagnosis and treatment are given according to the guidance provided by the Chinese National Health Commission. On March 11, 2020, the French government announced the launch of a clinical trial of treatment for pneumonia caused by SARS-CoV-2 to test the effectiveness of four different treatments. Inspired by the French clinical trial, we divided patients into four groups: the high-flow oxygen therapy group, the low-flow oxygen therapy group, the LPV/r group, and the interferon-alpha add LPV/r group.


**Ethics approval and consent to participate:** The study was approved by the Research Ethics Committee of Wuhan pulmonary Hospital and Xin Hua Hospital affiliated to Shanghai Jiao Tong University School of Medicine. Written informed consent was waived by the Ethics Commission of the designated hospital for this retrospective study. We have also certified that the study was strictly in accordance with the Declaration of Helsinki and International Ethical Guidelines for Health-related Research Involving Humans.

### 2.2 Data collection

Epidemiological, demographic, clinical, laboratory, treatment, and outcome data were extracted from electronic medical records. The data were reviewed by a trained team of physicians.

### 2.3 Definition

The date of disease onset was defined as the day when the symptom was noticed. Based on the epidemiological definition of the course, we did not simply take discharge as the endpoint of the survivor’s course, but for the first time the nucleic acid negative time after the patient’s symptoms improve (excluding the delay in hospitalization due to other diseases and the interval wait for discharge). For non-survivors, the end time of the course is the time of death. The course of COVID-19 was accurately recorded. According to the oxygen inhaled flow per minute, we divided oxygen inhalation therapy into low flow 1–3 L/min and high flow 4–8 L/min.

### 2.4 Statistical analysis

Continuous and categorical variables were presented as median (interquartile range) and *n* (%), respectively. A two-sided α of less than 0.05 was considered statistically significant. Statistical analyses were done using the SPSS (version 22.0). Cumulative rates of in-hospital death were determined using the Kaplan–Meier method. The risk factors and corresponding hazard ratios (HRs) were calculated using the Cox proportional hazard model.

## 3 Results

### 3.1 Baseline characteristics

We included 185 inpatients in the final analysis. Forty-three patients died during hospitalization and 142 were discharged. The median age was 62 years, the number of people in each age subgroup is 42 (22.7%) for <50 years, 128 (69.2%) for 50–70 years, and 15 (8.1%) for ≥75 years, and 50.8% were males. The median body mass index (BMI) was 22.96 and 119 (64.3%) cases were classified as severe (i.e., dyspnea, respiratory frequency ≥30/min, blood oxygen saturation ≤93%, partial pressure of arterial oxygen to fraction of inspired oxygen ratio <300, and/or lung infiltrates >50% within 24–48 h) at admission, among whom 81 (57%) survived and 38 (88.4%) died. Course of disease was 21 (16–24) days for survivors and 25 (18–31) days for non-survivors (*p* value = 0.001). More than half of the patients presented with fever (162 [87.6%]), dry cough (96 [51.9%]), and fatigue (98 [53.0%]). Other symptoms include chest stuffiness (70 [37.8%]), poor appetite (67 [36.2%]), sputum production (63 [34.1%]), shortness of breath (57 [30.8%]), myalgia (53 [28.6%]), diarrhea (35 [18.9%]), headache (31 [16.8%]), nausea and vomiting (21 [11.4%]), hemoptysis (12 [6.5%]), nasal congestion (10 [5.4%]), and pharyngalgia (7 [3.8%]). Patients with preexisting comorbidities conditions are as follows: 22.3% for diabetes, 37.8% for hypertension, 12.4% for cardiovascular disease, 13.5% for hyperlipemia, 3.8% for chronic obstructive pulmonary disease, 3.2% for chronic kidney disease, 4.3% for cerebrovascular disease, 4.9% for chronic liver disease, and 5.9% for cancer. The major complications of the cases include hypoproteinemia (53 [28.6%]), respiratory failure (39 [21.1%]), acute respiratory distress syndrome (33 [17.8%]), liver dysfunction (64 [34.6%]), acute kidney injury (10 [5.4%]), and electrolyte disturbances (59 [31.9%]). Of the 185 cases, 76 (41.1%) received high-flow oxygen therapy, 98 (53%) received low-flow oxygen therapy, 45 (24.3%) received noninvasive ventilation, 28 (15.1%) received invasive ventilation, 6 (3.2%) received ECMO, 12 (6.5%) received continuous renal replacement therapy, 162 (87.5%) received LPV/r, 141 (76.2%) received interferon-alpha add LPV/r, 170 (91.9%) received immune support, 127 (68.6%) received systemic corticosteroid, 88 (47.6%) received traditional Chinese medicine, 125 (67.6%) received budesonide atomization inhalation, and 60 (32.4%) received expectorant (Table 1).

**Table 1:** Baseline characteristics

	Total (*n*, %)	Survivor (*n*, %)	Non-survivor (*n*, %)	*p* value
	185	142	43	
Age, year	62.00 (50.00–68.00)	57.00 (49.00–65.75)	69.00 (64.50–72.00)	<0.001
Male	94 (50.8%)	64 (45.1%)	30 (69.8%)	0.005
Female	91 (49.2%)	78 (54.9%)	13 (30.2%)	
BMI	22.96 (21.05–25.30)	22.86 (20.70–25.29)	23.44 (21.77–25.33)	0.558
Smoking history	27 (14.6%)	16 (11.3%)	11 (25.6%)	0.020
History of surgery	53 (28.6%)	41 (28.9%)	12 (27.9%)	0.902
Drug allergy history	14 (7.6%)	10 (7%)	4 (9.3%)	0.742
Severe disease, %	119 (64.3%)	81 (57%)	38 (88.4%)	<0.001
Course of disease	21.00 (16.00–26.00)	21.00 (16.00–24.00)	25.00 (18.00–31.00)	0.001
**Signs and symptoms**				
Fever, >37.3℃	162 (87.6%)	122 (85.9%)	40 (93%)	0.216
Nasal congestion	10 (5.4%)	9 (6.3%)	1 (2.3%)	0.803
Fatigue	98 (53.0%)	75 (52.8%)	23 (53.5%)	0.938
Pharyngalgia	7 (3.8%)	7 (4.9%)	0 (0.0%)	0.204
Chest stuffiness	70 (37.8%)	51 (35.9%)	19 (44.2%)	0.185
Shortness of breath	57 (30.8%)	38 (26.8%)	19 (44.2%)	0.030
Cough	96 (51.9%)	71 (50%)	25 (58.1%)	0.349
Sputum production	63 (34.1%)	50 (35.2%)	13 (30.2%)	0.896
Hemoptysis	12 (6.5%)	8 (5.6%)	4 (9.3%)	0.478
Myalgia	53 (28.6%)	43 (30.3%)	10 (23.3%)	0.612
Headache	31 (16.8%)	27 (19.0%)	4 (9.3%)	0.135
Poor appetite	67 (36.2%)	45 (31.7%)	22 (51.2%)	0.020
Nausea and vomiting	21 (11.4%)	15 (10.6%)	6 (14.0%)	0.539
Diarrhoea	35 (18.9%)	24 (16.9%)	11 (25.6%)	0.203
Heart rate at admission, >100 bpm	76.00 (65.00–100.00)	76.00 (65.00–100.00)	78.00 (68.00–102.00)	0.226
Systolic blood pressure at admission, >130 mmHg	121.00 (111.50–133.50)	121.00 (113.00–133.00)	118.50 (107.50–137.25)	0.590
**Comorbidities**				
Any comorbidity	63 (34.1%)	45 (31.7%)	18 (41.9%)	0.218
Diabetes	41 (22.3%)	23 (16.3%)	18 (41.9%)	0.002
Hypertension	70 (37.8%)	42 (29.6%)	28 (65.1%)	<0.001
Cardiovascular disease	23 (12.4%)	11 (7.7%)	12 (27.9%)	<0.001
Hyperlipemia	25 (13.5%)	25 (17.6%)	0 (0.0%)	0.003
Chronic obstructive pulmonary disease	7 (3.8%)	4 (2.8%)	3 (7.0%)	0.210
Chronic kidney disease	6 (3.2%)	3 (2.1%)	3 (7.0%)	0.139
Cerebrovascular disease	8 (4.3%)	5 (3.5%)	3 (7.0%)	0.391
Tumor	11 (5.9%)	10 (7.0%)	1 (2.3%)	0.462
Chronic liver disease	9 (4.9%)	8 (5.6%)	1 (2.3%)	0.687
**Complications**				
Hypoproteinemia	53 (28.6%)	35 (24.6%)	18 (41.9%)	0.029
Respiratory failure	39 (21.1%)	13 (9.2%)	26 (60.5%)	<0.001
Acute respiratory distress syndrome	33 (17.8%)	8 (5.6%)	25 (58.1%)	<0.001
Liver dysfunction	64 (34.6%)	48 (33.8%)	16 (37.2%)	0.681
Electrolyte disturbances	59 (31.9%)	40 (28.2%)	19 (44.2%)	0.048
Acute kidney injury	10 (5.4%)	0 (0.0%)	10 (23.3%)	<0.001
**Laboratory findings**				
White blood cell count, ×10⁹/L	5.98 (3.91–9.54)	5.43 (3.69–8.57)	8.86 (5.37–12.93)	<0.001
<4	45 (46.9%)	40 (31.5%)	5 (12.5%)	<0.001
4–10	89 (53.3%)	70 (55.1%)	19 (47.5%)	
>10	33 (19.8%)	17 (13.4%)	16 (40%)	
Monocytes count, ×10⁹/L	0.32 (0.20–0.43)	0.31 (0.20–0.41)	0.34 (0.26–0.47)	0.094
>0.6	14 (8.4%)	10 (7.9%)	4 (10%)	0.745
Mononuclear cell ratio	5.45 (3.32–7.88)	5.87 (3.62–8.00)	4.00 (2.48–6.85)	0.014
<3	37 (22.3%)	22 (17.5%)	15 (37.5%)	0.017
3–10	116 (69.9%)	92 (73%)	24 (60%)	
>10	13 (7.8%)	12 (9.5%)	1 (2.5%)	
Lymphocyte count, ×10⁹/L	0.77 (0.55–1.19)	0.87 (0.59–1.25)	0.57 (0.45–0.75)	<0.001
<1.1	45 (27.1%)	42 (33.3%)	3 (7.5%)	0.001
Hemoglobin, g/L	128.00 (117.00–138.00)	128.00 (118.00–140.00)	127.00 (113.75–134.25)	0.158
<120	119 (72.1%)	91 (72.8%)	28 (70.0%)	0.731
Platelet, ×10⁹/L	184.50 (146.25–240.75)	192.00 (152.00–247.75)	152.00 (112.00–198.75)	0.006
<100	12 (7.2%)	5 (4%)	7 (17.5%)	0.015
100–350	148 (92.5%)	116 (95.9%)	32 (80%)	
>350	6 (3.6%)	5 (4%)	1 (2.5%)	
Eosinophil count, ×10⁹/L	0.00 (0.00–0.02)	0.00 (0.00–0.03)	0.00 (0.00–0.01)	0.032
<0.02	120 (72.3%)	85 (67.5%)	35 (87.5%)	0.014
Eosinophil ratio	0.10 (0.00–0.30)	0.10 (0.00–0.57)	0.00 (0.00–0.10)	0.001
<0.4	126 (75.9%)	89 (70.6%)	37 (92.5%)	0.005
Neutrophil number, ×10⁹/L	4.61 (2.71–7.84)	3.77 (2.43–6.31)	7.82 (5.03–11.60)	<0.001
>6.3	56 (33.7%)	32 (25.4%)	24 (60%)	<0.001
High-sensitivity C-reactive protein, mg/L	35.92 (16.17–85.91)	32.42 (14.06–80.58)	74.20 (28.12–100.40)	0.002
>5	144 (85.7%)	106 (82.2%)	38 (97.4%)	0.017
**Treatment**				
High flow oxygen therapy	76 (41.1%)	58 (40.8%)	18 (41.9%)	0.906
Low flow oxygen therapy	98 (53.0%)	89 (62.7%)	9 (20.9%)	<0.001
**Mechanical ventilation**				
Noninvasive	45 (24.3%)	13 (9.2%)	32 (74.4%)	<0.001
Invasive	28 (15.1%)	2 (1.4%)	26 (60.5%)	<0.001
Continuous renal replacement therapy	12 (6.5%)	1 (0.7%)	11 (25.6%)	<0.001
ECMO	6 (3.2%)	0 (0.0%)	6 (14.0%)	<0.001
Immune support	170 (91.9%)	134 (94.4%)	36 (83.7%)	0.025
Expectorant	60 (32.4%)	33 (23.2%)	27 (62.8%)	<0.001
Budesonide atomization inhalation	125 (67.6%)	95 (66.9%)	30 (69.8%)	0.725
Traditional Chinese medicine	88 (47.6%)	76 (53.5%)	12 (27.9%)	0.003
Systemic corticosteroid	127 (68.6%)	96 (67.6%)	31 (72.1%)	0.578
LPV/r	162 (87.5%)	125 (88%)	37 (86.0%)	0.730
Interferon add LPV/r	141 (76.2%)	112 (78.9%)	29 (67.4%)	0.123

### 3.2 Association of treatments with in-hospital death

**Figure 1:**
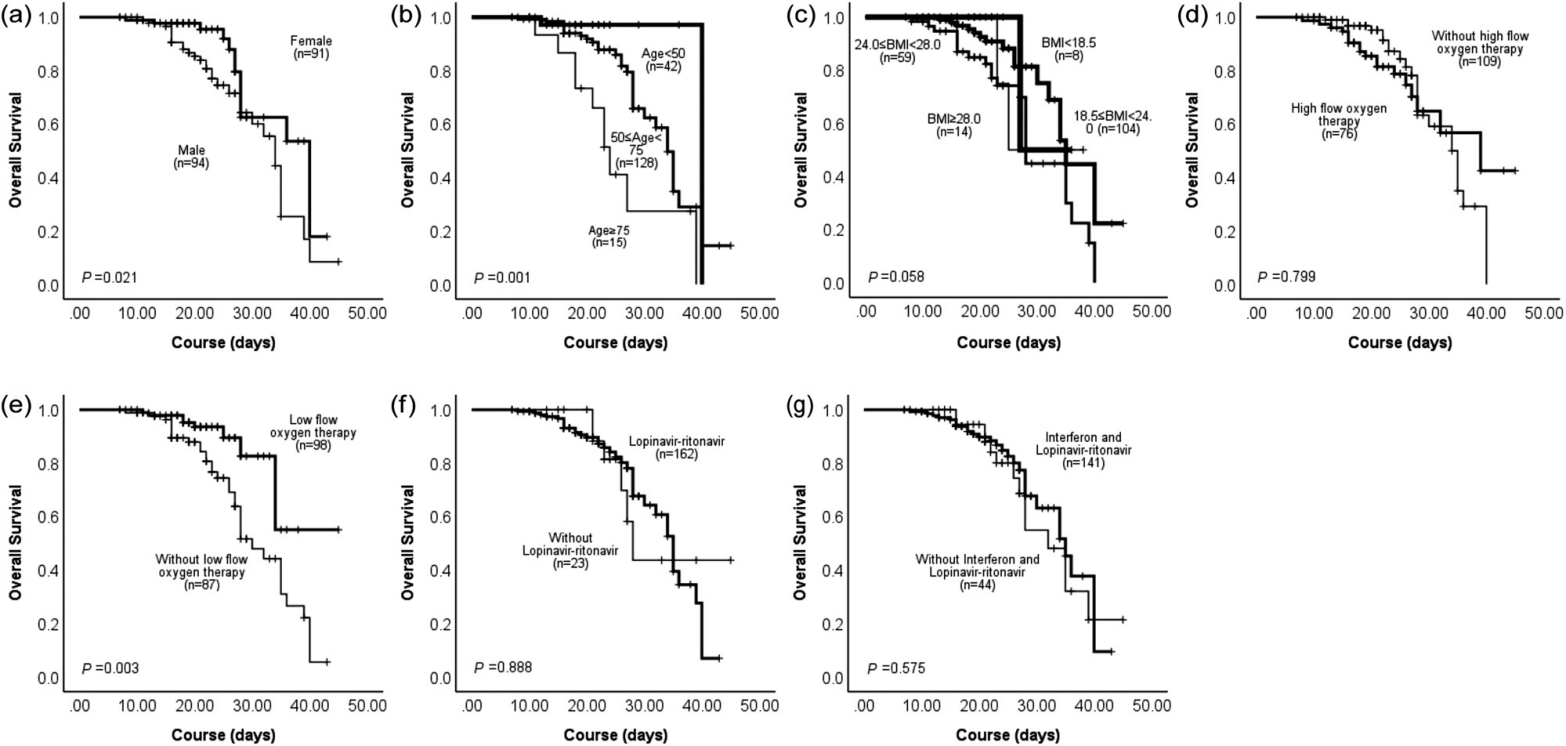
Kaplan–Meier analysis demonstrated that mortality was higher in older patients (*p* = 0.001), higher in men than in women (*p* = 0.021), and lower in the low-flow oxygen therapy group than in the non-low-flow oxygen therapy group. BMI had no influence on mortality (*p* = 0.058), as well as high flow oxygen therapy, LPV/r therapy, and interferon-alpha add LPV/r therapy.

The in-hospital death occurred in 43 (23.2%) patients in our study. Kaplan–Meier analysis demonstrated that mortality was higher in older patients (*p* = 0.001), higher in men than in women (*p* = 0.021), and lower in the low-flow oxygen therapy group than in the non-low-flow oxygen therapy group. BMI had no influence on mortality (*p* = 0.058), as well as high flow oxygen therapy, LPV/r therapy, and interferon-alpha add LPV/r therapy (Figure 1). *p* values of each group are as follows: high-flow oxygen therapy group (*p* = 0.799), low-flow oxygen therapy group (*p* = 0.003), the LPV/r group (*p* = 0.888), and the interferon-alpha add LPV/r group (*p* = 0.575). Cox proportional hazard regression confirmed that the low-flow oxygen therapy was an independent protective factor for in-hospital death after adjusting for age, gender, and BMI (Table 2): the high-flow oxygen therapy group (HR: 0.909, 95% CI: 0.479–1.724, *p* = 0.770), the low-flow oxygen therapy group (HR: 0.369, 95% CI: 0.170–0.801, *p* = 0.012), the LPV/r group (HR: 0.798, 95% CI: 0.252–2.524, *p* = 0.701), the interferon-alpha add LPV/r group (HR: 1.556, 95% CI: 0.641–3.777, *p* = 0.328), gender (HR: 2.105, 95% CI: 1.031–4.295, *p* = 0.041), age (HR: 1.084, 95% CI: 1.042–1.127, *p* < 0.001), and BMI (HR: 1.093, 95% CI: 0.979–1.219, *p* = 0.114).

**Table 2:** Cox proportional hazard regression confirmed that the low-flow oxygen therapy was independent protective factor for in-hospital death after adjusting for age, gender, and BMI

	All-cause mortality	
	HR (95% CI)	*p* value
Gender (male vs female)	2.105 (1.031–4.295)	0.041
Age (years)	1.084 (1.042–1.127)	<0.001
BMI	1.093 (0.979–1.219)	0.114
High flow oxygen therapy	0.909 (0.479–1.724)	0.770
Low flow oxygen therapy	0.369 (0.170–0.801)	0.012
LPV/r	0.798 (0.252–2.524)	0.701
Interferon add LPV/r	1.556 (0.641–3.777)	0.328

## 4 Discussion

In this retrospective study, 185 patients with confirmed COVID-19 admitted to Wuhan Pulmonary Hospital from January 1 to February 29, 2020, were enrolled. We selected several types of antiviral drugs that are now clinically concerned and applied to clinical treatment and divided subjects into two groups: the LPV/r group and the interferon-alpha add LPV/r group. In addition to the above two treatment methods, we studied the correlation between high-flow oxygen therapy and low-flow oxygen therapy with in-hospital mortality of COVID-19 patients. Results showed that mortality was higher in older patients, higher in men than in women, and lower in the low-flow oxygen therapy group than in the non-low-flow oxygen therapy group. BMI had no influence on mortality, as well as high-flow oxygen therapy, LPV/r therapy, and interferon-alpha add LPV/r therapy. The low-flow oxygen therapy was independent protective factor for in-hospital death after adjusting for age, gender, and BMI. LPV/r is a human immunodeficiency virus (HIV) protease inhibitor approved for HIV-1 infection patients. It is recommended by the “Guidelines for diagnosis and treatment of novel coronavirus pneumonia (Trial Version 5)” for the treatment of patients with COVID-19 [6]. Our results confirmed that there is no statistically significant on the mortality of COVID-19 between patients with or without LPV/r treatment. The latest results of Professor Cao Bin’s team in China also indicated that the treatment of LPV/r is ineffective for COVID-19 [7]. Interferon is a biological response regulator. Interferon has the effect of antiviral enhancement of immunity and is widely used in clinical antiviral therapy. The combination of interferon and LPV/r is often used in the clinical antiviral therapy, but in this study, the treatment of interferon-alpha add LPV/r had no effect on hospitalization mortality in patients with COVID-19. The study gave the conclusions that the mortality was lower in the low-flow oxygen therapy group than in the group without using low-flow oxygen therapy, and the mortality of inpatients with COVID-19 was not affected by high-flow oxygen therapy. It is not difficult to understand that patients with low-flow oxygen therapy were less ill, so the prognosis is better. In addition, our study found that mortality was higher in older patients, higher in men than in women. BMI had no influence on mortality. The same conclusion has been drawn from many existing studies [8–12]. Because advanced age is an independent risk factor and older patients may have multi-system underlying diseases and a decline in autoimmunity, both will lead to high mortality after suffering from COVID-19. Many studies on the clinical characteristics of COVID-19 have also confirmed that the mortality of COVID-19 in male patients is higher than that in female patients. BMI is an important international measure of obesity and health but had no effect on mortality in this study. There were some limitations in this study. First, this is a single-center retrospective observational study with limitations of study number and region; thus, the multicenter and prospective studies should be performed to further clarify the correlation between drug therapy and the mortality rate of COVID-19 inpatients. Second, the number of COVID-19 case in this study is still less; the large sample study is more likely to reduce the potential bias of the sample itself. Although we tried to adjust for many confounders, there may exist confounders either unmeasured or unknown that could explain our observed results.

## 5 Conclusions

The mortality was higher in older patients, higher in men than in women, and lower in the low-flow oxygen therapy group than in the non-low-flow oxygen therapy group. BMI had no influence on mortality, as well as high flow oxygen therapy, LPV/r therapy, and interferon-alpha add LPV/r therapy. The low-flow oxygen therapy was an independent protective factor for in-hospital death after adjusting for age, gender, and BMI.

## Abbreviations

BMI: body mass index
CI: confidence interval
COVID-19: coronavirus disease 2019
HR: hazard ratio
SARS-Cov-2: severe acute respiratory syndrome coronavirus 2
WHO: World Health Organization

## References

[1] Chinese researchers reveal draft genome of virus implicated in Wuhan pneumonia outbreak. (https://www.sciencemag.org/news/2020/01/chinese-researchers-reveal-draft-genome-virus-implicated-wuhan-pneumonia-outbreak).

[2] Zhu N, Zhang D, Wang W, Li X, Yang B, Song J, et al. A novel coronavirus from patients with pneumonia in China, 2019. N Engl J Med. 2020;382:727–33. 10.1056/NEJMoa2001017.Epub2020. PMC7092803 31978945

[3] WHO Director-General’s Remarks at the Media Briefing on 2019-nCoV on 11 February 2020. (https://www.who.int/dg/speeches/detail/who-director-general-s-remarks-at-the-media-briefing-on-2019-ncov-on-11-february-2020).

[4] Li Q, Guan X, Wu P, Wang X, Zhou L, Tong Y, et al. Early transmission dynamics in Wuhan, China, of novel coronavirus-infected pneumonia. N Engl J Med. 2020;382(13):1199–207. 10.1056/NEJMoa2001316.Epub2020. PMC7121484 31995857

[5] Holshue ML, DeBolt C, Lindquist S, Lofy KH, Wiesman J, Bruce H, et al. First case of 2019 novel coronavirus in the United States. N Engl J Med. 2020;382(10):929–36. 10.1056/NEJMoa2001191.Epub2020. PMC7092802 32004427

[6] Zeng YM, Xu XL, He XQ, Tang SQ, Li Y, Huang YQ, et al. Comparative effectiveness and safety of ribavirin plus interferon-alpha, lopinavir/ritonavir plus interferon-alpha and ribavirin plus lopinavir/ritonavir plus interferon-alphain in patients with mild to moderate novel coronavirus pneumonia. Chin Med J. 2020;133(9):1132–4. 10.1097/CM9.0000000000000790. PMC7213617 32149772

[7] Cao B, Wang Y, Wen D, Liu W, Wang J, Fan G, et al. A Trial of Lopinavir–Ritonavir in adults hospitalized with severe Covid-19. N Engl J Med. 2020;382(19):1787–99. 10.1056/NEJMoa2001282.Epub2020. PMC7121492 32187464

[8] Mo P, Xing Y, Xiao Y, Deng L, Zhao Q, Wang H, et al. Clinical characteristics of refractory COVID-19 pneumonia in Wuhan, China. Clin Infect Dis. 2020;73(11):e4208–213. 10.1093/cid/ciaa270. PMC7184444 32173725

[9] Wang C, Pan R, Wan X, Tan Y, Xu L, Ho C, et al. Immediate psychological responses and associated factors during the initial stage of the 2019 coronavirus disease (COVID-19) epidemic among the general population in China. Int J Environ Res Public Health. 2020;17(5):1729. 10.3390/ijerph17051729. PMC7084952 32155789

[10] Wenham C, Smith J, Morgan R. COVID-19: the gendered impacts of the outbreak. Lancet (London, Engl). 2020;395:846–8. 10.1016/s0140-6736(20)30526-2.Epub2020. PMC7124625 32151325

[11] Zhao S, Cao P, Chong MK, Gao D, Lou Y, Ran J, et al. The time-varying serial interval of the coronavirus disease (COVID-19) and its gender-specific difference: A data-driven analysis using public surveillance data in Hong Kong and Shenzhen, China from January 10 to February 15, 2020. Infect Control Hospital Epidemiol. 2020;10(5):1–8. 10.1017/ice.2020.64. PMC7113032 32146921

[12] Wu J, Liu J, Zhao X, Liu C, Wang W, Wang D, et al. Clinical characteristics of imported cases of COVID-19 in Jiangsu Province: a multicenter descriptive study. Clin Infect Dis. 2020;71(15):706–12. 10.1093/cid/ciaa199. PMC7108195 32109279

